# Supplementary material for: The Nature of the Spark Is a Pivotal Element in the Design of a Miller–Urey Experiment
Source: Life (Basel). 2023 Nov 12;13(11):2201. doi: 10.3390/life13112201 (PMC10672138; doi:10.3390/life13112201)
Supplement: Supplementary file 1 [file life-13-02201-s001.zip › life-2665698-supplementary.pdf]

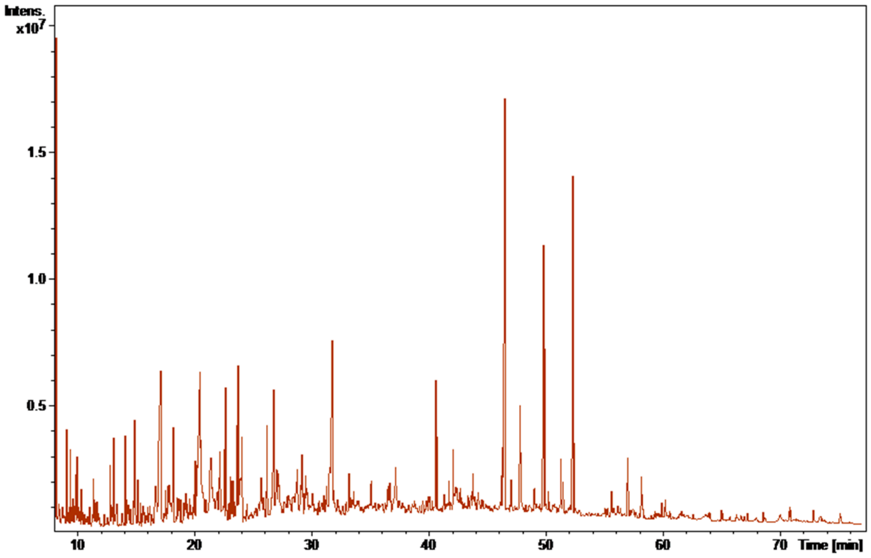

Figure S1. The TIC chromatogram of the miller soup volatile compounds obtained from GC-MS

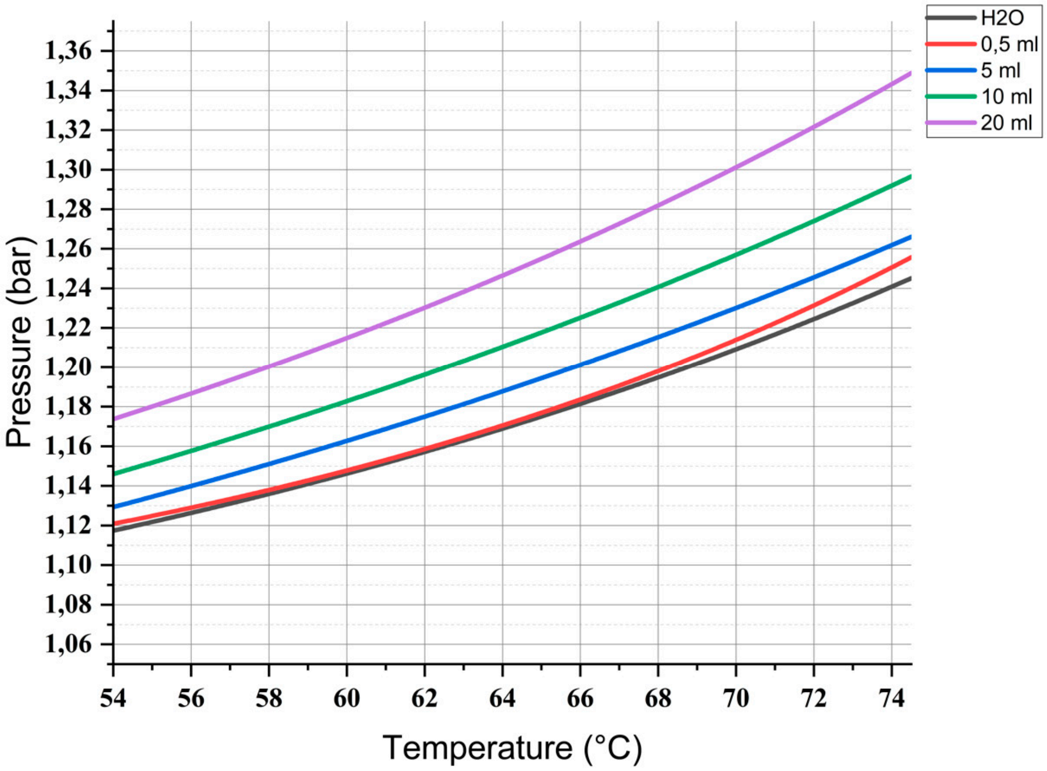

Figure S2. Pressure in the reactor as a function of temperature for different added amounts of 35% ammonia/water solution (see insert, H2O means no ammonia added). Pressure corresponds the total pressure within the reactor in equilibrium. Temperature corresponds to the actual temperature in the middle of the reactor. (P_{atm} = 0.9896 Bar, T = 22 ± 2 °C)

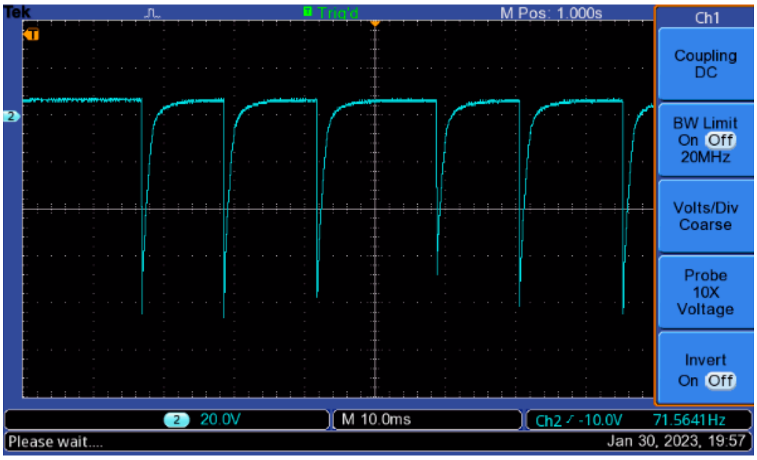

Figure S3. Voltage-time characterization of FB-1

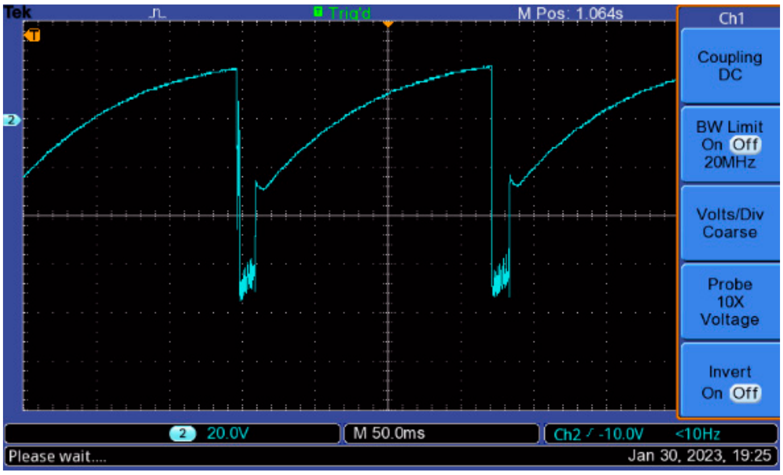

Figure S4. Voltage-time characterization of FB-2

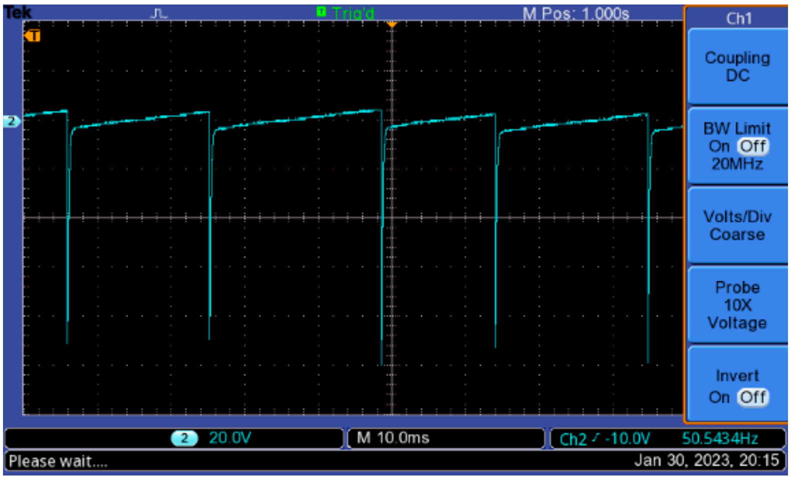

Figure S5. Voltage-time characterization of CA-1

Figures S.3-S5. Voltage as a function of time for spark generators FB-1, FB-2 and CA-1. The sudden breakdowns in tension correspond to spark generation.

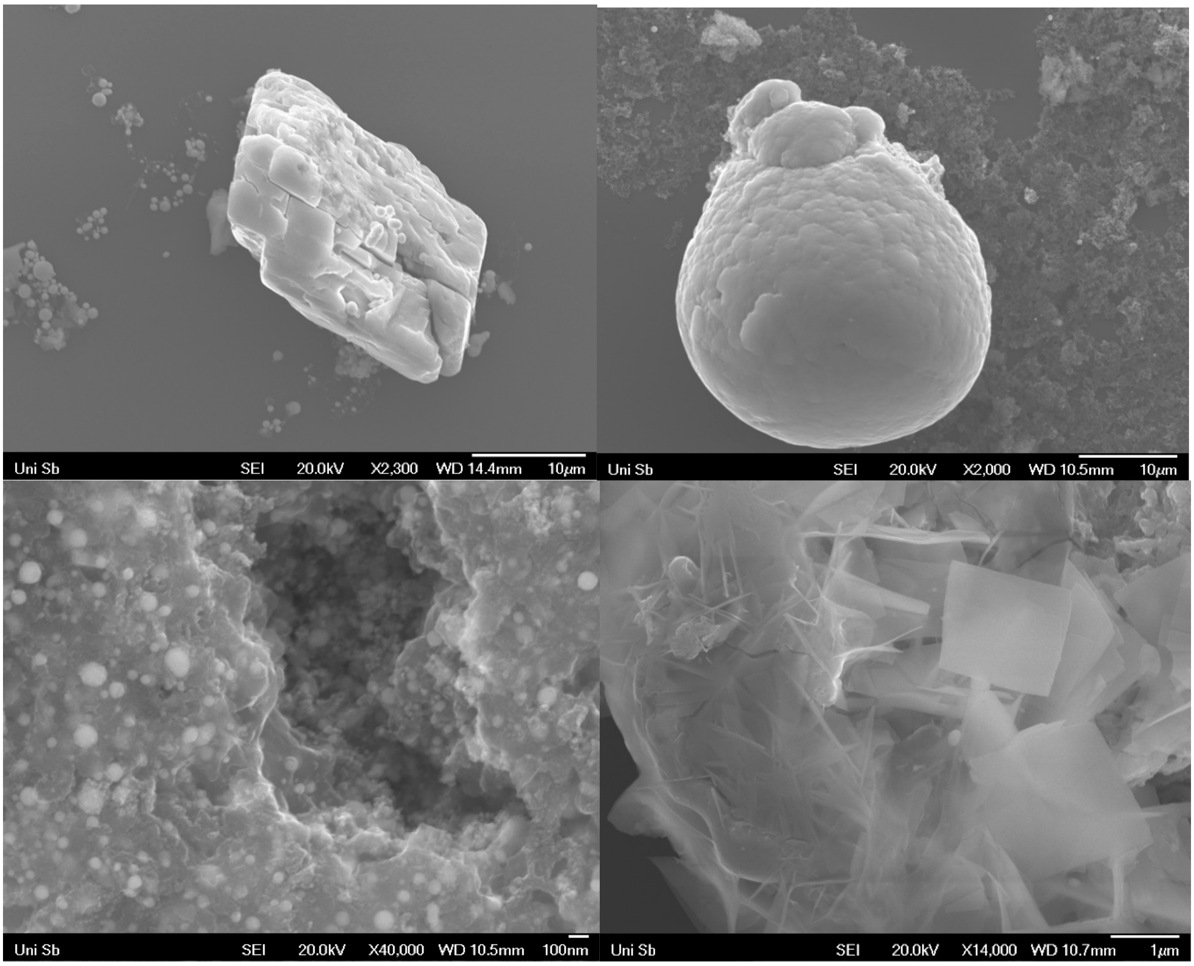

Figure S6. SEM pictures of Black material

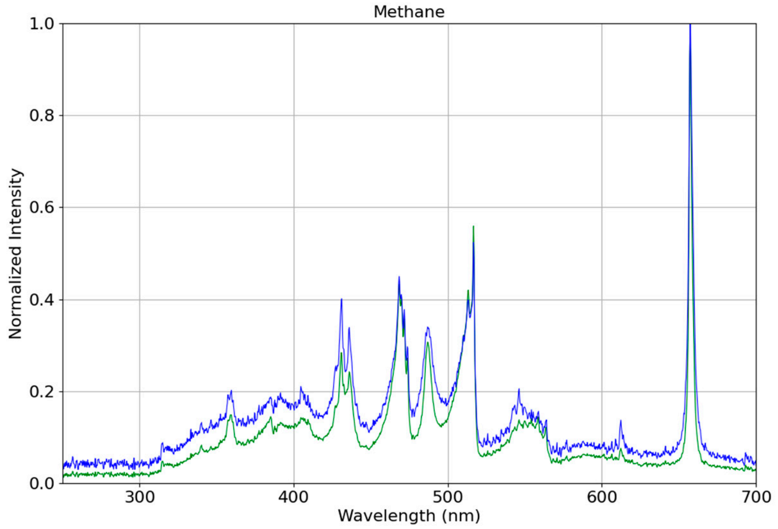

Figure S7. The methane excitation spectrum of spark generators FB-1 (blue) and FB-2 (green).

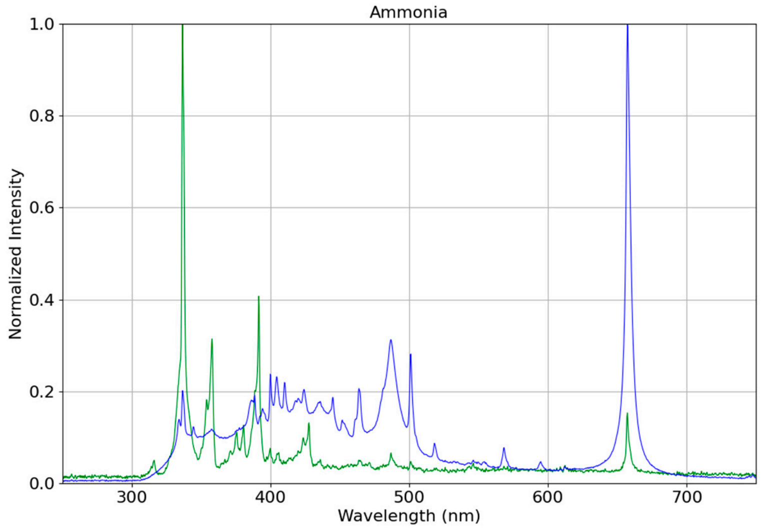

Figure S8. The ammonia excitation spectrum of spark generators FB-1 (blue) and FB-2 (green).

Table S1. The 20 most abundance fragments as observed by GC/MS

Experiment #	Fragments abundance decrease (m/z) →																			
Sample 1	**73**	**117**	**75**	**132**	**313**	**129**	**57**	**221**	**43**	**145**	**147**	**71**	**341**	**355**	**85**	**55**	**281**	**131**	**41**	**69**
Sample 2	**117**	**73**	**75**	**132**	**313**	**129**	**57**	**43**	**145**	**71**	**341**	**55**	**85**	**41**	**131**	**69**	**185**	**314**	**133**	**118**
Sample 3	**117**	**73**	**75**	**132**	**313**	**129**	**57**	**43**	**145**	**71**	**341**	**85**	**55**	**41**	**131**	**69**	**314**	**133**	**118**	**201**
Sample 4	**73**	**221**	**75**	**117**	**222**	**263**	**57**	**43**	**147**	**132**	**71**	**129**	**313**	**223**	**74**	**133**	**85**	**145**	**205**	**131**
Sample 5	**73**	**165**	**75**	**147**	**180**	**117**	**221**	**330**	**175**	**45**	**129**	**43**	**74**	**114**	**128**	**166**	**132**	**257**	**100**	**243**
Sample 6	**165**	**73**	**180**	**75**	**166**	**149**	**175**	**117**	**147**	**43**	**57**	**45**	**221**	**74**	**71**	**132**	**98**	**181**	**129**	**167**
Sample 7	**117**	**73**	**341**	**75**	**132**	**129**	**145**	**57**	**43**	**55**	**342**	**131**	**71**	**226**	**69**	**133**	**41**	**85**	**201**	**118**
Sample 8	**73**	**165**	**147**	**75**	**117**	**180**	**43**	**45**	**149**	**166**	**131**	**74**	**129**	**59**	**330**	**221**	**116**	**57**	**71**	**103**

Table S2. Spark generator electrical characteristics

**Spark generators**	FB-1	FB-2	CA-1
**V input**	19.00 V	15.00 V	11.50 kV
**I input**	0.100 A	0.500 A	0.107 mA
**V Output (kV)**	9	10	11
**Sparking frequency (Hz)**	71	10	50

Table S3. Number of assigned compounds by GC/MS

Experiment #	Number of assigned compounds
**Sample 1**	140
**Sample 2**	154
**Sample 3**	351
**Sample 4**	261
**Sample 5**	330
**Sample 6**	124
**Sample 7**	305
**Sample 8**	160

Table S4. Assigned compounds by GC/MS

Sub-molecular class affiliation examples	Assigned structure	Prob.	NIST N.
**Amines**	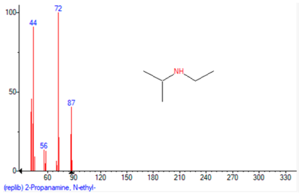	70.0	77935
	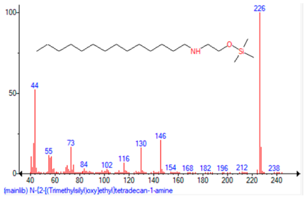	10.4	350495
	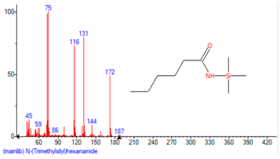	83.7	408867
	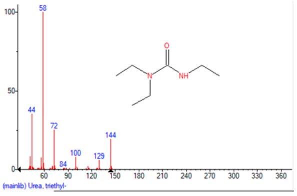	82.9	96499
	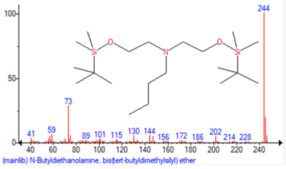	54.9	53512
	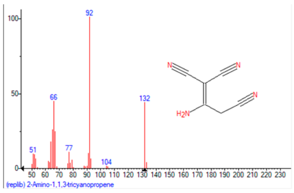	68.5	112363
	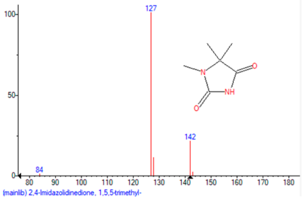	37.6	62814

	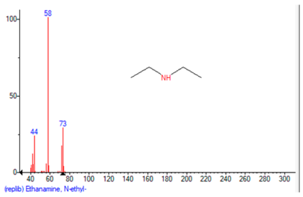	32.3	108362
	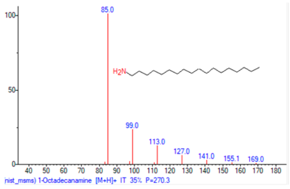	50.2	95529
	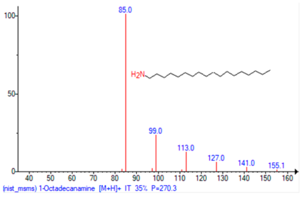	51.7	9374
	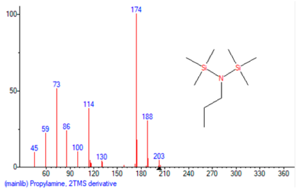	13.9	213850
**Urea**	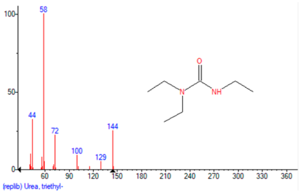	76.7	103776
**Fatty acids**	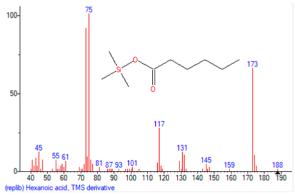	75.2	299366
	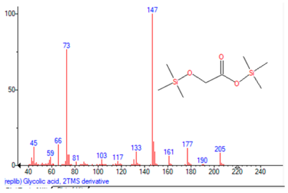	86.6	123654
	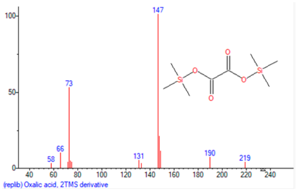	34.1	32847

	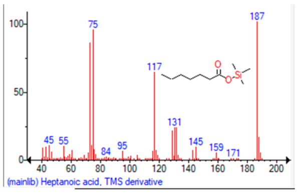	96.9	226308
	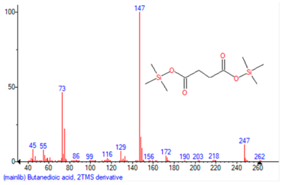	38.7	719422
	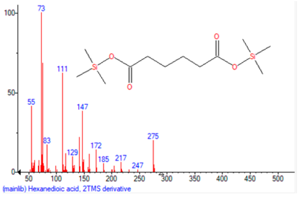	78.7	78965
	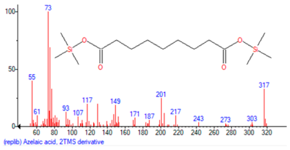	75.9	36218
	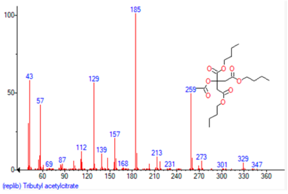	93.2	6856862
**Fatty alcohols**	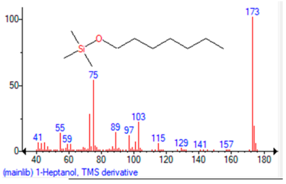	88.5	196061
	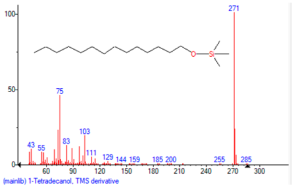	64.8	207261
	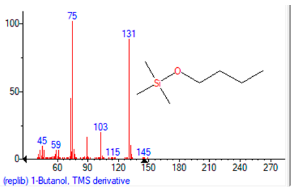	29.7	1227418

	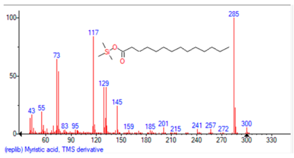	62.7	510592
	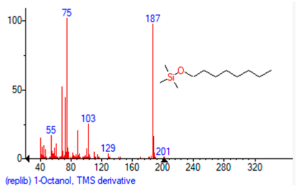	74.1	385924
	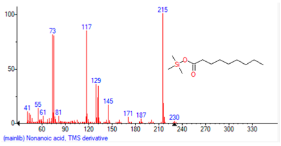	98.5	379255
	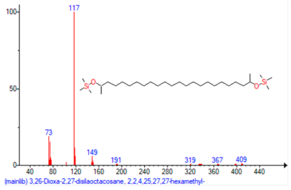	10.1	94899
	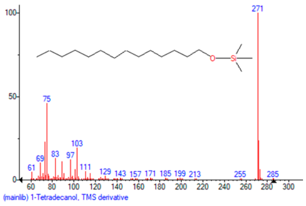	25.1	73173
	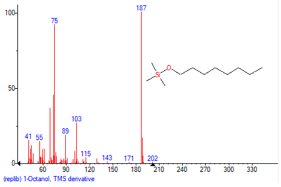	75.0	406733
	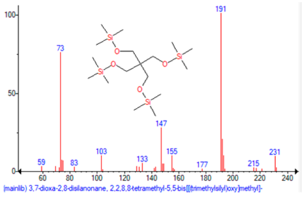	59.4	98774
	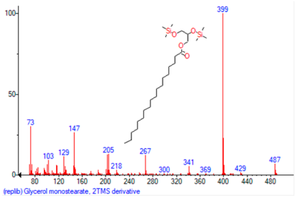	47.0	57600

	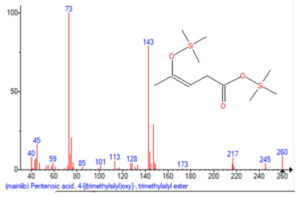	64.8	237792
**Aromatics**	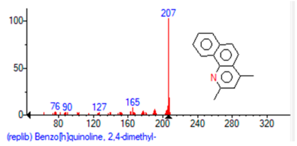	16.2	17819
	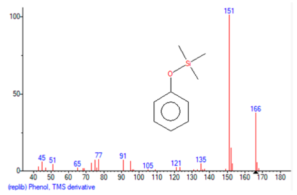	80.2	760495
	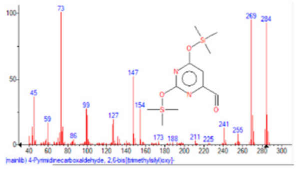	69.5	24956
	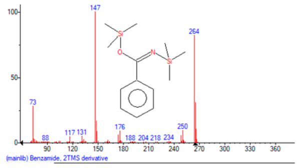	96.7	417798
	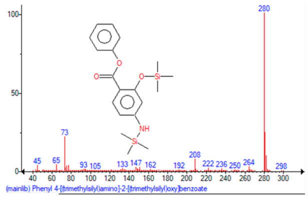	53.3	166876
	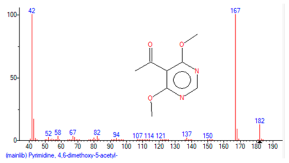	98.3	74292
	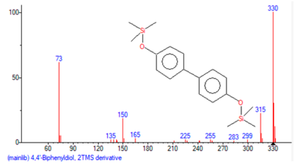	82.1	15405
	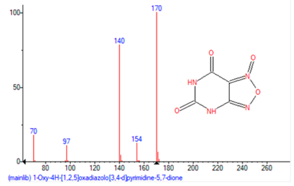	63.4	71420

	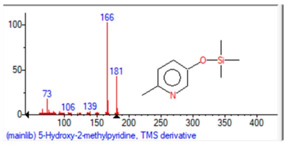	36.7	59448
	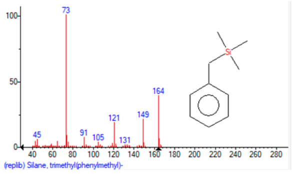	44.10	71467
	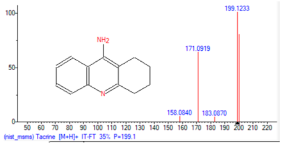	26.0	78787
	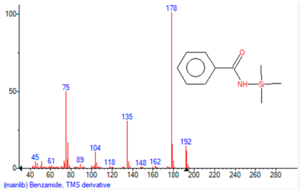	92.8	339015
	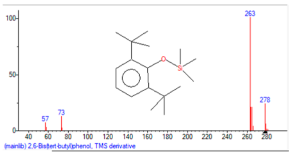	80.4	624192
	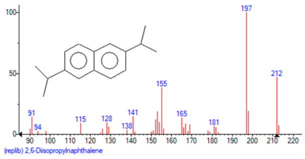	48.6	26593
	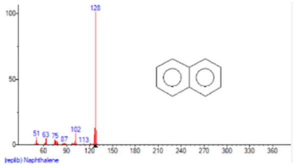	47.5	379701
	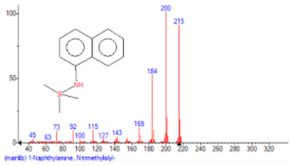	94.8	417695
	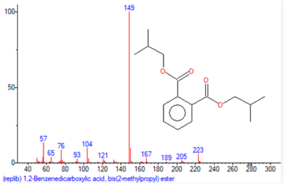	12.8	814787

	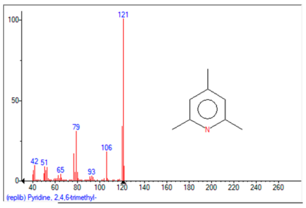	96.3	1719783
	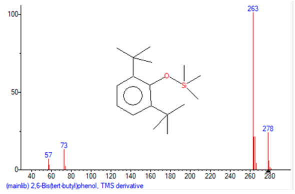	81.1	686938
	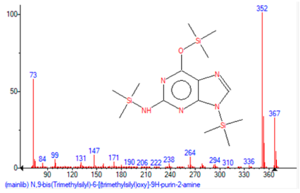	84.8	189227
	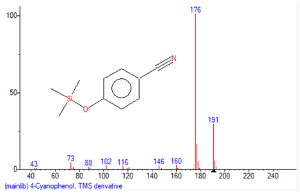	93.8	1376725
	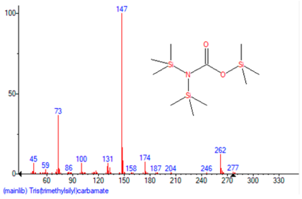	79.8	2170001
	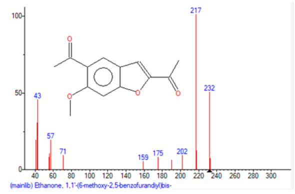	71.7	143403
	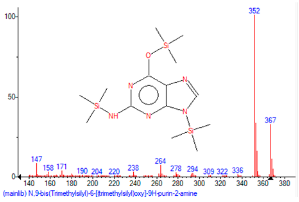	80.0	28854
	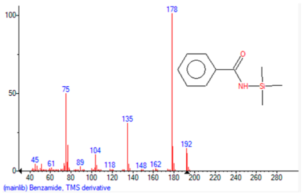	92.8	339015

	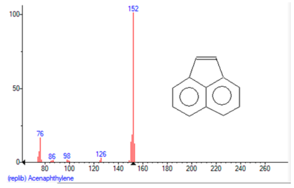	70.3	36439
	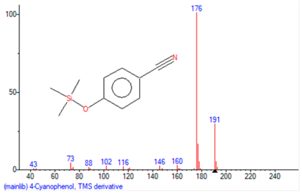	42.8	4644168
	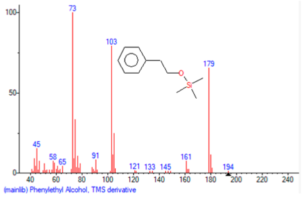	89.0	1041722
	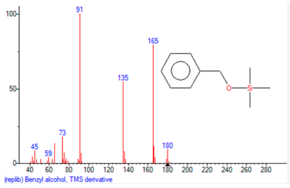	75.0	374100
	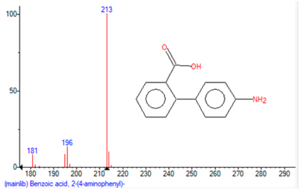	57.7	65246
	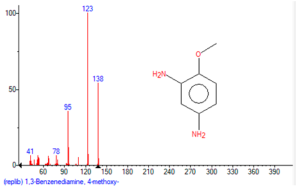	36.4	160871
	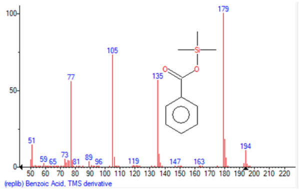	68.0	87963
	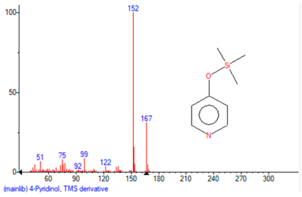	71.9	756386

	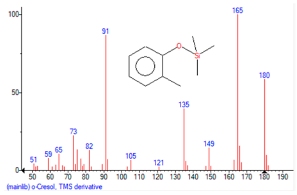	66.4	23903
	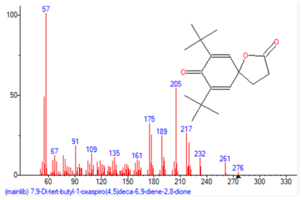	90.9	60506
**PAHs**	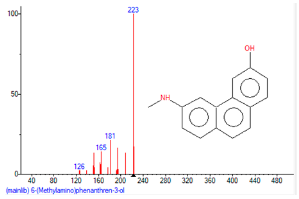	29.7	103705
	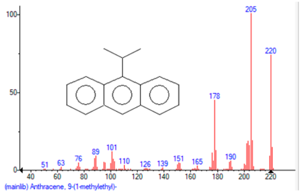	16.6	115574
	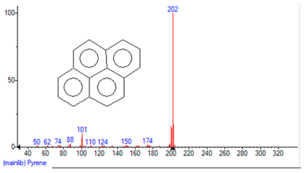	65.9	227992
	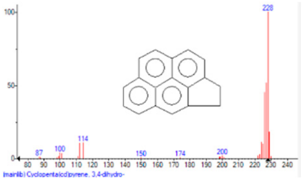	71.9	74738
	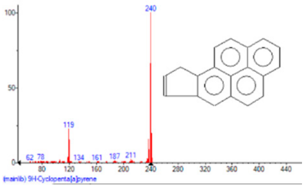	97.3	326095
	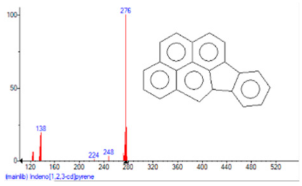	60.7	114983

	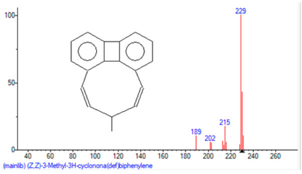	86.6	147978
	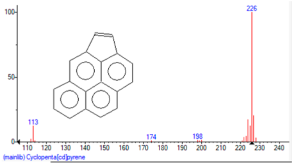	60.1	74739
	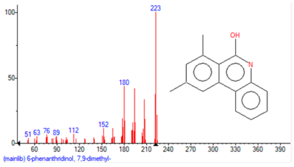	95.4	396294
	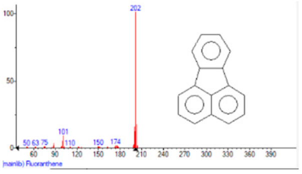	72.5	228362
	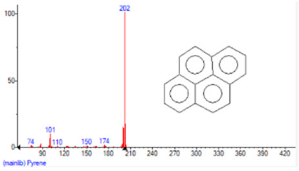	41.4	227992
**Amides**	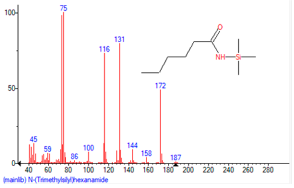	48.3	289197
	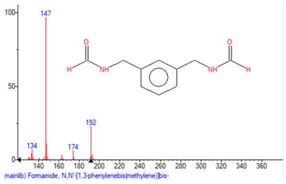	43.5	202417
**Amino acids**	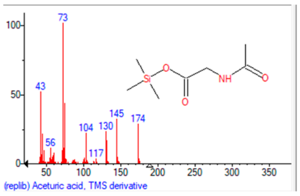	40.5	275909
	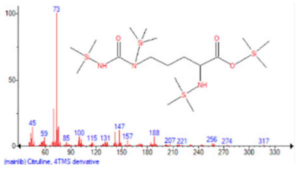	31.4	17846

	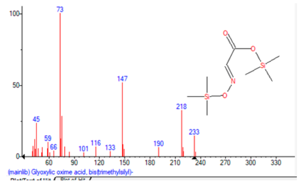	29.7	78864
	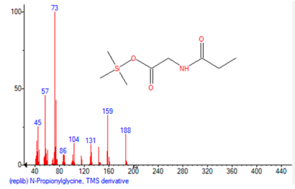	31.0	960881
	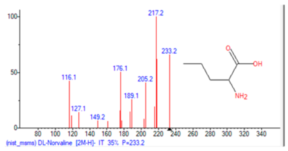	20.0	21164
	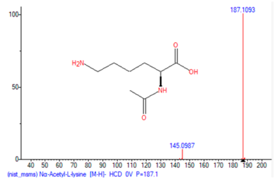	71.1	186737
	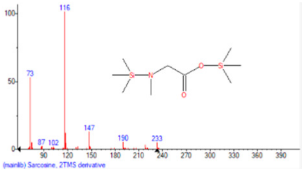	26.1	153975
	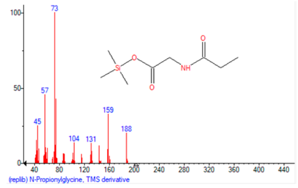	40.3	835375
	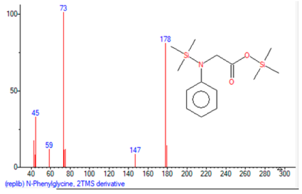	30.1	484429
	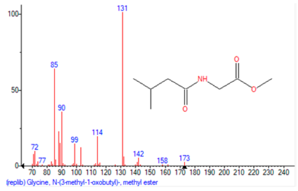	35.4	111238
	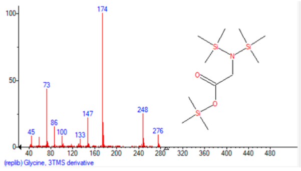	35.2	158658

**Saturated hydrocarbons**	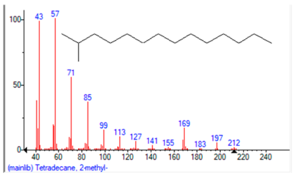	19.3	707968
	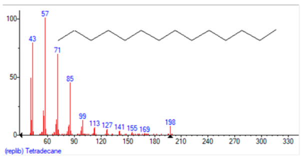	35.5	1589508
	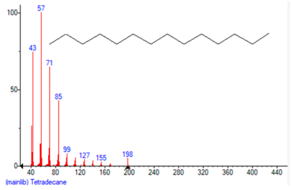	30.3	1950172
	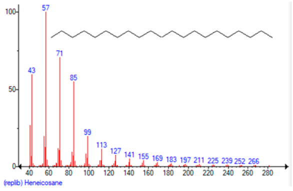	8.14	4520627
	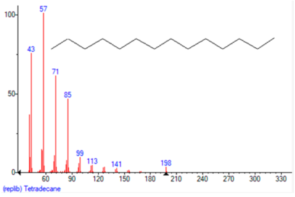	43.1	1527630
	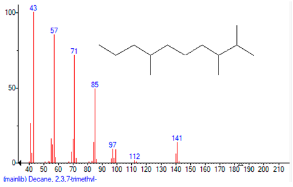	62.4	17267
	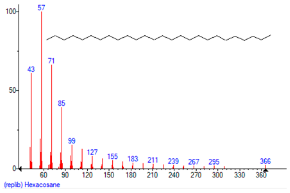	21.8	304116
	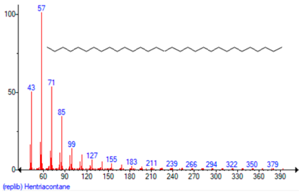	15.0	654619

	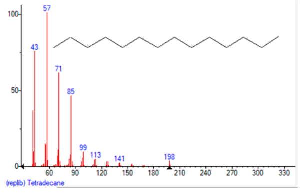	41.9	1506272
**Others**	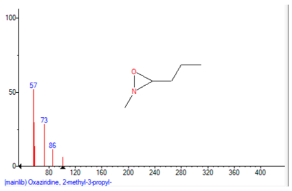	31.1	18533
	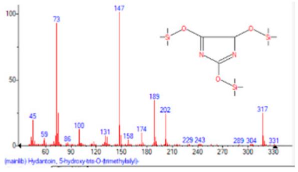	80.9	149425
	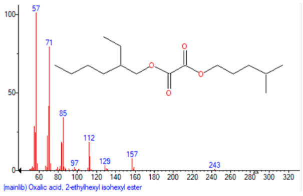	12.5	85426
	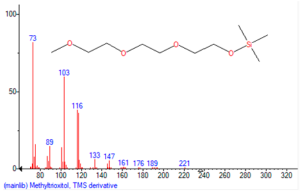	35.4	11325
	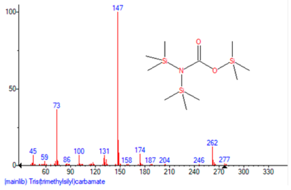	86.4	8057243
	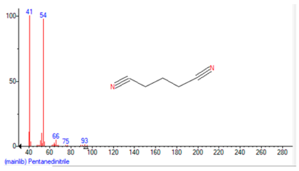	87.1	2330325
	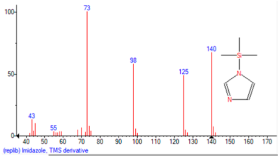	94.0	161540

	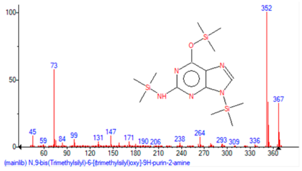	77.7	333943
	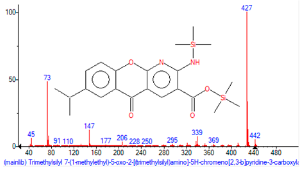	54.3	408271
	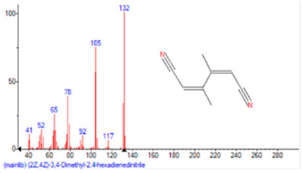	69.5	210603
	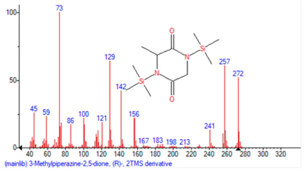	80.0	333700

Note: PAHs refer to polycyclic Aromatic Hydrocarbons.
